# Early Recovery of Exercise-Related Muscular Injury by HBOT

**DOI:** 10.1155/2019/6289380

**Published:** 2019-05-29

**Authors:** Chen-Yu Chen, Wen-Yi Chou, Jih-Yang Ko, Mel S. Lee, Re-Wen Wu

**Affiliations:** ^1^Department of Orthopedic Surgery, Kaohsiung Chang Gung Memorial Hospital, Taiwan; ^2^Graduate Institute of Clinical Medical Science, Chang Gung University College of Medicine, Kaohsiung, Taiwan

## Abstract

Early recovery from muscular injury is crucial for elite athletes. Hyperbaric oxygen therapy (HBOT) has been reported to be beneficial in terms of accelerating cell recovery and tissue repair, which are considered to be helpful for eliminating fatigue and recovering stamina. This study was performed to evaluate the efficacy of HBOT for exercise-related muscular injury. Forty-one athletes with exercise-related muscular injuries were recruited and randomized into an HBOT group and a control group. All participants received 10 sessions of either HBOT or placebo treatment. The brief pain inventory (BPI) was completed, and serum samples were analyzed. Data were collected before treatment (T1), at the end of the fifth treatment session (T2), at the end of the tenth treatment session (T3), and two weeks after T3 (T4). At T3, the HBOT group showed prominent reductions in the levels of creatine phosphokinase (CK), glutamic oxaloacetate transaminase (GOT), and myoglobin (MB), which lasted until T4. However, the control group did not present any statistical differences in levels from T1 to T4. In terms of pain intensity and interference, the HBOT group showed significant improvements at T3, while no improvements were observed in the control group. In conclusion, HBOT facilitates the early recovery of exercise-related muscular injury. This trial is registered with ISRCTN17817041.

## 1. Introduction

Muscular strains are the most common type of muscle injury sustained during participation in highly competitive sports [1, 2]. Performance reduction and increased risks of severe sports injuries, such as ligamentous injury, tendon tears, or fractures, have been reported to be related to adjacent muscular injury [3, 4]. Although the best treatment strategies for muscular injury remain controversial, popular treatment modalities include rest, ice, compression, bandaging, physical therapy to improve the joint's range of motion, and medications (pain-control or anti-inflammatory agents), which require several weeks to months of treatment to achieve complete recovery [5]. However, early recovery from muscular injury is crucial for elite athletes who are regularly exposed to high-stress training and competition. Therefore, an alternative to conventional treatment is desired to shorten the recovery time.

Hyperbaric oxygen therapy (HBOT) is a safe, effective, and noninvasive treatment that has been applied to treat various conditions [6]. During HBOT, the patient is placed in a hyperbaric chamber, which pressurized to 1.4 atmospheres absolute (ATA) or higher and is supplemented with pure oxygen [7]. This promotes the proliferation and differentiation of endogenous stem cells and suppresses inflammation, to achieve clinical effects [8, 9]. By increasing the partial pressure of oxygen in tissues, HBOT can also result in vasoconstriction, angiogenesis, and fibroblast proliferation, enhance the aggregation of red blood cells toward oxidation, and improve the distribution of oxygen in tissues [10–16]. Accordingly, HBOT has been reported to be beneficial in accelerating cell recovery and tissue repair, which are considered to help eliminate fatigue and recover stamina. HBOT has gained considerable attention among sports medicine specialists as an adjuvant therapy to accelerate athletes' muscular injury recovery, but the exact efficacy remains unclear [8, 17–20].

Many studies have assessed the extent of muscle tissue damage by measuring the levels of creatine phosphokinase (CK), myoglobin (MB), glutamic oxaloacetate transaminase (GOT), and other muscle-cell proteins in the blood stream [21–26]. These enzymatic changes in the blood reflect muscle activity and are recognized as catabolic muscular enzymes that can be used to monitor muscular injury and catabolism [23]. In this study, a prospective, randomized, double-blind clinical control trial was conducted to clarify the efficacy of HBOT for muscular injury recovery. The effects were assessed in elite athletes via serum levels of catabolic and muscular enzymes and the brief pain inventory (BPI), which included measurements of pain intensity and pain interference. We hypothesized that HBOT could facilitate the early recovery of exercise-related muscular injury and could therefore be beneficial for elite athletes.

## 2. Materials and Methods

This prospective, randomized, double-blind control study was approved by the Institutional Review Board (IRB) of Chang Gung Medical Foundation (IRB Study No. 102-2994B). Written informed consent was obtained from all study participants. The study complies with the ethical standards for scientific research on sports and exercise [27].

This clinical trial recruited 46 adult baseball players who had sustained prolonged (more than 2 weeks) exercise-induced muscular soreness or pain with grade I muscle strain of the extremities. The muscular strain type I was diagnosed by the history of an acute painful event during the competition or training and characterized by the mild swelling, local tenderness on the muscle belly, such as quadriceps, hamstring, calf muscles, biceps, and triceps. The regional X ray was taken to exclude the bony lesion if necessary. The athletes were regularly involved in intensive sports activities for at least 4 hours a day, 4 days per week, and 9 months per year. Owing to the minor muscular strain without significant functional disability, the athletes in this study kept training or competition as their routine schedule before and during the HBOT study which contained at least four days of intensive training or competition in a week. The continuous high tensile sports activities retard or result in the inadequate recovery from the minor muscular strain. To minimize the comparative bias of HBOT efficacy in different degrees or patterns of injuries, the athletes were excluded from the study if they sustained a new-onset injury during the treatment, including grade II or III muscular strain or ligamentous sprain. Other exclusion criteria included claustrophobia, pneumothorax, asymptomatic air cysts or blebs in the lungs, infection of the upper respiratory tract, recent facial contusion, and a history of chest or ear surgery.

**Figure 1:**
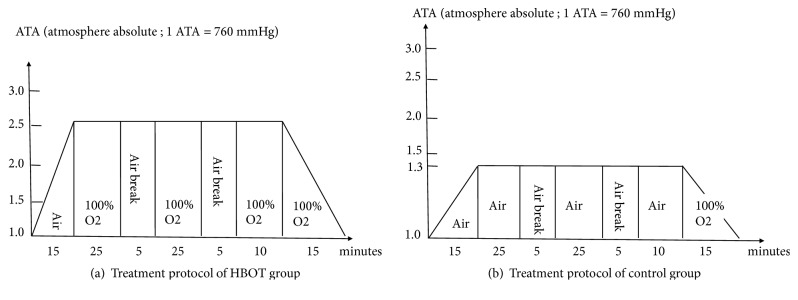
(a) Treatment protocol of the HBOT group. (b) Treatment protocol of the control group.

The participants were equally divided into an HBOT group and a control group using a computer-based, randomized, and double-blind allocation method. The enrolled athletes were all blinded to the allocation. The interviewer who administered the questionnaire was also blinded to the study allocation, and the concealed allocation was revealed two weeks after the treatment session was completed. The allocation was also concealed to all participants. Athletes who were assigned to the HBOT group were placed in a hyperbaric chamber that was pressurized to 2.5 ATA over 15 min and then supplied with pure oxygen for 25 min, followed by a 5-min air break. This cycle was repeated once, followed by a cycle of pure oxygen for 10 min, during which the chamber was depressurized to 1 ATA over 15 min while supplying 100% oxygen (Figure 1(a)).

The athletes assigned to the control group were subjected to a similar treatment cycle, but the hyperbaric chamber was pressured to 1.3 ATA over 15 min, and 100% oxygen was substituted with natural air. 100% oxygen was only supplied for 15 min during depressurization of the hyperbaric chamber (Figure 1(b)). The duration of each session was 100 minutes. The participants in both groups received two sessions per week for five weeks.

Venous serum samples were harvested before the first session (T1), at the end of the fifth session (T2), at the end of the tenth session (T3), and two weeks after the end of the tenth session (T4). The analysis of these samples included the measurement of blood urea nitrogen (BUN), creatine phosphokinase (CK), lactate (LT), glutamic oxaloacetate transaminase (GOT), and myoglobin (MB). The participants were asked to complete the BPI before the first session (T1) and at the end of the tenth session (T3). The BPI is a structured questionnaire on pain intensity and pain interference that has been proven to have high reliability [28, 29]. The BPI provides the rapid assessment of severity of pain, the impact on functioning, and emotional status. It contained the severity of pain, interference of pain on daily function, mood, sleep, and relations with other people in last week. The primary outcome was defined as the immediate efficacy of the HBOT in terms of both serum markers and BPI at the end of 10 sessions.

PASS 15.0 was adopted to estimate the appropriate sample size. Group sample sizes of 16 and 16 would achieve 81% power to detect a difference of 12.9 between the null hypothesis that both group means would be 34.3 and the alternative hypothesis that the mean of group 2 would be 21.4. This was determined with known group standard deviations of 14.8 and 9.5 and a significance level (alpha) of 0.05 using a two-sided Mann-Whitney test, assuming that the actual distribution is normal.

The data were processed using SPSS software version 22.0 (SPSS Inc., Chicago, IL, USA). Participants' demographics, blood results, and BPI scores were represented as mean values, standard deviations, and percentages. Finally, an independent t-test, chi-squared test, Fisher's exact test, Pearson's product–moment correlation coefficient analysis, and the Generalized Estimating Equation (GEE) model were used to analyze the differences in metabolic indices and the effectiveness of treatment in the two groups before, during, and after intervention and during the follow-up period.* p* values less than 0.05 were regarded as indicating statistical significance.

## 3. Results

**Figure 2:**
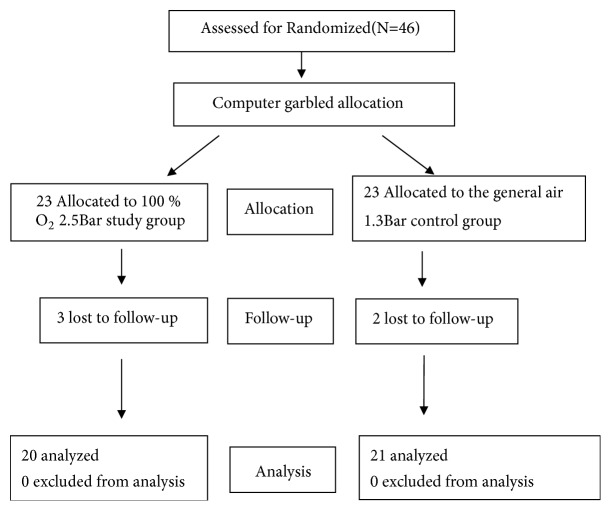
Flowchart of participants throughout the comparison of the HBOT and control groups.

Forty-six athletes were initially enrolled and randomized equally into the HBOT group and control group. Following the exclusion of subjects with incomplete data and those lost to follow-up, 20 athletes remained in the HBOT group, and 21 remained in the control group for the final analysis (Figure 2). There were no significant differences in demographic variables between the two groups (Table 1).

**Figure 3:**
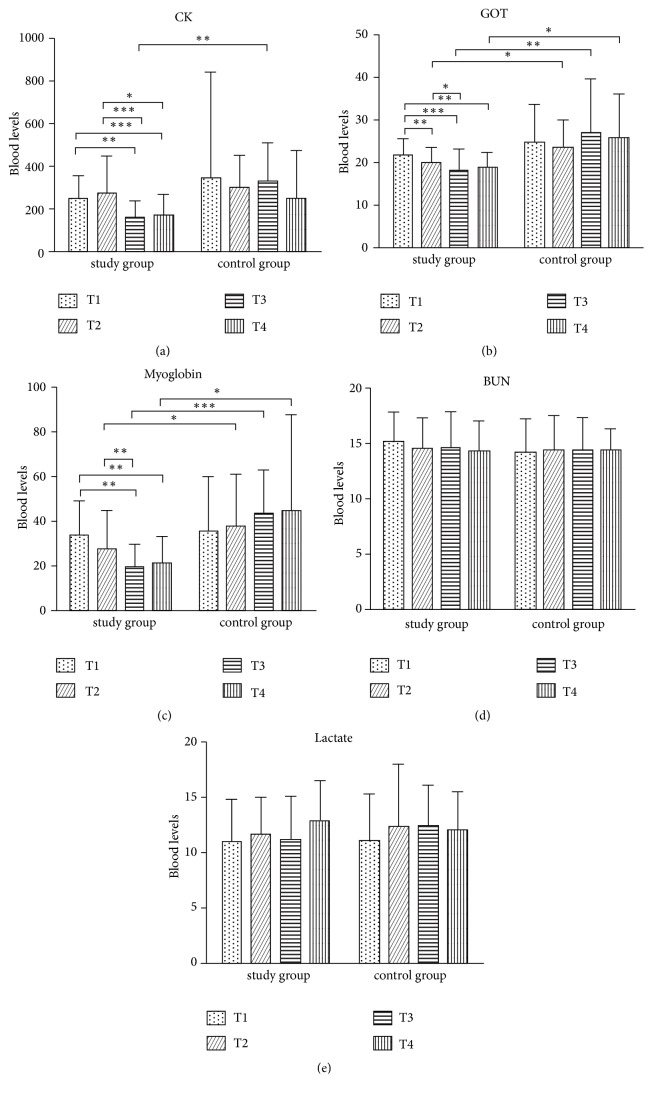
Comparative outcomes of serum catabolic and muscular enzyme levels. BUN:mg/dl; CPK:U/L; LT: mg/dl; GOT: U/L; MB: ng/ml. *∗*p < 0.05, *∗∗*p < 0.01, *∗∗∗*p < 0.001.

Professional baseball players accounted for 56% of the participants, and the remaining were college players. Regarding play positions, 68.2% were pitchers, and 31.8% were positional players. In addition, 4.8% of the players had a history of surgery that was performed at least 1 year prior to this study. At T1 (before the intervention), no differences were found in the BUN, LT, CK, GOT, and MB concentrations of the participants in the HBOT group and control group (Figure 3).

### 3.1. Effects of HBOT on Serum Catabolic and Muscular Enzymes

At T1, both groups were not significantly different in terms of CK, GOT, MB, BUN, and LT (Table 2). The HBOT group showed significant reductions of CK (p < 0.001) at T3 (Figure 3(a)) and in GOT (p = 0.004) and MB (p < 0.001) from T2 to T4 compared to the control group (Figures 3(b) and 3(c)). We found early improvements in GOT in the HBOT group at T2, and the effect of HBOT in these muscular enzymes lasted until two weeks after the HBOT. However, neither the HBOT group nor the control group exhibited significant changes in the levels of BUN and LT. The improvement in muscular enzymes indicated positively effects of HBOT on the recovery of muscular injury.

### 3.2. Effects of HBOT on the Brief Pain Inventory (Pain Intensity and Pain Interference)

At T1, there were no significant differences regarding the 11 items of the BPI between groups (Table 3). In the 4 items of the pain intensity subscale, the HBOT group revealed significant improvement at T3 in comparison with the control group. The HBOT group also revealed significant differences in these 4 items when comparing T1 to T3, but the control group did not. Regarding the 7 items of the pain interference subscale, there were also no significant differences between groups at T1. Compared with the control group, the HBOT group showed significant improvement in 5 of the 7 subscales but did not exhibit differences in “Walking ability” and “Normal work.” In comparison with T1, the HBOT group demonstrated significant improvement in all items except “Relations with other people,” and the control group only showed significant differences in “Mood,” “Normal work,” and “Sleep.”

## 4. Discussion

When training or competing in high-intensity sports, the stress or injuries that athletes experience often cause physical and mental exhaustion and anxiety and influence their sleep and mood. The present results showed that HBOT significantly reduced muscular enzyme levels and improved the pain intensity and interference in almost every subscale, which are beneficial for highly competitive athletes. Another study enrolled 60 participants to use a cycle ergometer to exhaustion and showed that those in the HBOT group exhibited a lower lactate concentration that those in the control group (without HBOT intervention). This suggested that HBOT alleviated the extent of lactate elevation caused by exercise [25]. However, there was no improvement in serum lactate in the present study, which is compatible with other research indicating that exercise-induced lactate elevation is not influenced by HBOT [30]. In the present study, all participants were elite baseball players who frequently suffered exercise-related muscle injuries. The discrepant results may have been caused by the different methods of energy exertion examined in the studies or the different types of athletes recruited.

The present results also indicated that the BUN concentration remained unchanged in both groups throughout the entire research period (T1–T4). These results were also consistent with a study that analyzed samples of fingertip blood from 14 table tennis players to determine the change in BUN concentration before and after training [31]. According to a literature review and the present results, the effects of HBOT on the improvement of serum catabolic enzymes are inconclusive.

Horie et al. demonstrated that HBOT improved the force-producing capacity of regenerating muscle fibers and accelerated satellite cell proliferation and myofiber maturation in muscular injury in rats [8]. It effectively eliminates the accumulation of fatigue-inducing substances and regulates metabolism [8]. Moreover, HBOT can increase the oxygen supply to the tissue, energize cell activity, promote adenosine triphosphate (ATP) synthesis, and facilitate the metabolism of fatigue-inducing substances. Based on these proposed effects, HBOT was applied in the treatment of muscular damage under fatigue [8].

The extent of muscular damage can be evaluated by analyzing the blood protein concentrations of CK, MB, and other muscle cell proteins [3, 4, 19, 25, 32]. The previous study demonstrated the ranges of serum GOT (21.5 ± 4.5 U/L), CK (175.8 ± 80.7 U/L) [33], and MB (50.2 ± 6.7 ng/ml) level in the athletes with muscular strain, which were within the range of both HBOT and control group of the present study. The present study revealed that HBOT reduced the CK level at the end of treatment sessions compared to the control group (Table 2, Figure 3(a)). It significantly improved the CK concentration in the HBOT group by 35.4% at T3, while the control group did not show any improvement. However, the CK results were inconsistent with several previous studies. One of the studies measured the CK concentration in 21 college students after they engaged in 10 minutes of forearm flexor exercises. The results revealed that HBOT failed to improve the CK concentration [34].

Another study measured the CK concentration in 16 university students after they completed a single-leg eccentric exercise task involving the quadriceps femoris. The CK concentration in the study group improved while that in the control group increased rapidly. However, the two groups did not present significant differences (*p*=0.31) [35]. We postulated that these discrepancies were due to the length and intensity of the exercise, and the use of HBOT to alleviate athlete fatigue is a viable option that requires further research.

Regarding GOT, there were significant differences between the two groups starting at T2, and favorable effects were evident in the HBOT group at T3 (Table 2, Figure 3(b)). The HBOT group exhibited significant improvement at T2 to T4, with increases of 8.3%, 17%, and 12.6% during each period. In contrast, the control group exhibited an improvement of 1.38% at T2, which was not statistically significant (*p*>0.05). A previous study reported that the GOT concentration in 25 rug by players attending a 20-day camp significantly increased due to strenuous daily exercise [11]. These results are consistent with the findings of the present study, suggesting that individuals who are involved in daily strenuous exercise are prone to sustaining musculoskeletal fatigue and increased muscle cell membrane permeability, which induce the release of GOT enzymes and cause the accumulation of fatigue [24].

MB is an iron- and oxygen-binding protein in muscle tissue, and high concentrations reflect specific muscular damage. A significant reduction was noted in the MB level in the HBOT group at T2 (19.5%), T3 (42.7%), and T4 (37.2%) in comparison with T1. In contrast, the control group showed an increasing trend from T2 to T4 (Figure 3(c)). A significant difference in the MB level was observed between the two groups at T3 (*p*>0.05), suggesting that the muscular damage in the control group was not effectively controlled. HBOT significantly reduced the MB level from T2 to T4, which indicated benefits of the treatment in terms of recovery of muscular damage. Based on the present results for CK, GOT, and MB, we concluded that HBOT is beneficial for the reduction of serum intracellular muscular enzymes, which implies a positive effect on the early recovery of muscular damage.

There is controversy in the literature regarding the effectiveness of HBOT for symptom relief. A review of the literature revealed that HBOT effectively alleviates muscle soreness associated with sports injuries [8, 19–21, 35, 36]. However, other studies have concluded that no substantial evidence was obtained to indicate that HBOT effectively alleviates or treats ankle sprains, acute knee ligament injuries, experiment-induced delayed-onset muscle soreness, or exercise-induced muscle injury [31, 34, 37–39]. In the present study, a different assessment modality for pain was adopted for the precise analysis of pain reduction in the HBOT group and control group. The BPI was used to measure the average pain in the high-intensity athletes and its extent. This method is more specific and precise than the conventional visual analogue scale.

The pain experienced by the participants in the HBOT group was effectively reduced following T3 (*p*<0.001), which proves that HBOT is a feasible means of improving exercise-induced muscular injury. The present results were consistent with those of a number of studies, such as HBO treatment accelerated myofiber maturation in rat muscular injury [8], and improve the recovery of delayed-onset muscle soreness in the quadriceps [31, 35, 40]. Therefore, administering HBOT after exercise can promote the recovery of muscle recovery and postulated to be helpful for the muscular pain regression [8]. The pain interference results indicated that all pain interference items were alleviated after the intervention except for “relations with other people.” Therefore, the results showed that HBOT effectively improved pain intensity and pain interference.

There were various limitations in this study. First, the ideal interval between each HBOT session remains unclear. Limiting the sessions to two per week might have weakened the efficacy in comparison to daily administration. However, in light of the burden of regular training, daily administration of HBOT is not feasible for professional athletes. Second, the dosages used for HBOT at each session are not well-defined, such as the duration or the amount of 100% O_2_ delivery. The employed treatment protocol was a routine protocol rather than a specialized regimen for the treatment of sports injuries. Third, muscular injury has not been officially approved as an indication for HBOT by the Food and Drug Administration. This highlights the need for further study regarding the appropriate application and dosages of HBOT.

Sports-induced muscle injury can cause muscle failure, soreness, swelling, stiffness, and reduced muscle strength [4, 41]. It also alters the concentrations of hormones secreted by the endocrine glands and cells in the blood. An athlete's competitive performance is correlated with their recovery ability and the amount of fatigue-inducing substances in their bloodstream. Based on a literature review and the present results, we concluded that HBOT could be a viable alternative to current common treatment protocols to facilitate the early recovery of exercise-related muscular injury.

## 5. Conclusion

HBOT was found to successfully reduce pain interference, which improved the participants' general activity, mood, walking ability, sleep, normal work, and enjoyment of life. It also reduced the serum levels of CK, GOT, and MB, which could be an indication of recovery of muscular injury. In conclusion, HBOT could be a safe and effective treatment modality that facilitates early recovery of exercise-related muscular injury in elite athletes.

## Figures and Tables

**Table 1:** Demographic characteristics of the athletes.

		HBOT Group		Control Group		*p-value*
		(*n*=20)		(*n*=21)		
		*n*	%	*n*	%	
Gender	male	20		21		0.218
Age (years)		23.9 ± 5.1		26.3 ± 5.6		0.254
Level of play						0.310^*F*^
	professional	11	52	12	57	
	amateur	10	48	9	43	
Defensive position						0.658
	pitcher	13	65	15	71.4	
	fielder	7	35	6	28.6	
Internal diseases						0.410^*F*^
	Yes	4	20	2	9.5	
	No	16	80	18	90.5	
Surgical history						1.000^*F*^
	Yes	1	5	1	4.8	
	No	19	95	19	95.2	

*Note*. M: Mann-Whitney U test, *F*: Fisher's exact test, else Chi-square test.

**Table 2:** Comparative outcomes in serum enzymatic changes.

Variable	HBOT (n=20)	Control (n=21)	P-value^1^
	Mean ± SD	Mean ± SD	
CPK(U/L)			
T1	245.4 ± 109.8	342.1 ± 496.8	0.404
	(166–1167)	(82–289)	
T3	158.5 ± 78.0	327.1 ± 181.1	0.001
	(62–275)	(152–796)	
P-value^2^	0.005	0.908	

GOT(U/L)			
T1	21.8 ± 3.9	24.9 ± 8.8	0.402
	(18–45)	(11–30)	
T3	18.2 ± 5.0	27.1 ± 12.6	0.004
	(13–32)	(12–38)	
P-value^2^	<0.001	0.483	

Myoglobin (ng/mL)			
T1	33.9 ± 15.4	35.6 ± 24.3	0.584
	(20.5–94.5)	(12.6–39.1)	
T3	19.4 ± 10.2	43.3 ± 19.4	<0.001
	(13–17.5)	(14.9–84.7)	
P-value^2^	0.001	0.280	

BUN (mg/dL)			
T1	15.2 ± 2.7	14.2 ± 3.1	0.188
	(10–20)	(10–16)	
T3	14.6 ± 3.3	14.5 ± 2.9	0.858
	(9–15)	(10–17)	
P-value^2^	0.371	0.640	

Lactate (mg/dL)			
T1	11.0 ± 3.9	11.1 ± 4.3	0.958
	(5.1–20.8)	(5.4–19)	
T3	11.2 ± 4.0	12.5 ± 3.7	0.163
	(6.5–18.6)	(8.3–36)	
P-value^2^	0.893	0.123	

*∗*Mann-Whitney U test was used to compare 2 groups

*∗* Generalized Estimating Equation (GEE) model was used to compare T1-T3

*∗*P-value^1^: Comparison of data between HBOT group and Control group

P-value^2^: Comparison of data between T1 and T3.

Note: The laboratory normal value of these blood markers: BUN: 6–21 mg/dl; CPK: 56–226 U/L; LT: 4.5–19.8 mg/dl; GOT: 0–37 U/L; MB: 17.4–105.7 ng/ml.

**Table 3:** Comparative outcomes of pain intensity and pain interference.

Variable	HBOT Group	Control Group	P-value^1^
	(*n*=20)	(*n*=21)	
	*Mean* ± *SD*	*Mean* ± *SD*	
Pain at its worst in the last week			
T1	4.9 ± 1.5	3.95 ± 2.2	0.173
T3	2.4 ± 1.6	4.0 ± 1.8	0.002
P-value^2^	0.005	0.640	
Pain at its least in the last week			
T1	4.3 ± 1.5	3.5 ± 2.0	0.135
T3	1.5 ± 1.7	3.3 ± 1.8	0.002
P-value^2^	<0.001	0.688	
Pain on average in the last week			
T1	4.6 ± 1.5	3.5 ± 1.9	0.119
T3	1.8 ± 1.6	3.5 ± 1.6	0.001
P-value^2^	<0.001	0.909	
Pain right now			
T1	4.6 ± 1.6^c^	3.7 ± 2.0	0.160
T3	1.40 ± 1.7	3.3 ± 1.8	0.001
P-value^2^	<0.001	0.346	
Pain interference in the last week (1)			
General activity			
T1	1.9 ± 1.0	2.2 ± 2.4	0.566
T3	0.8 ± 1.6	2.1 ± 2.4	0.040
P-value^2^	0.005	0.653	
Pain interference in the last week (2) Mood			
T1	2.7 ± 1.1	2.1 ± 1.9	0.171
T3	1.1 ± 1.6	3.6 ± 1.8	<0.001
P-value^2^	<0.001	0.001	
Pain interference in the last week (3) Walking ability			
T1	1.2 ± 0.9	1.3 ± 1.8	0.476
T3	0.7 ± 1.6	1.2 ± 2.0	0.580
P-value^2^	0.005	0.653	
Pain interference in the last week (4)			
Normal work continued			
T1	1.4 ± 0.9	1.3 ± 1.7	0.302
T3	0.8 ± 1.3	1.4 ± 1.7	0.254
P-value^2^	<0.001	0.001	
Pain interference in the last week (5)			
Relations with other people			
T1	1.8 ± 0.8	1.5 ± 1.6	0.221
T3	0.7 ± 1.4	2.2 ± 1.5	<0.001
P-value^2^	0.161	0.847	
Pain interference in the last week (6)			
Sleep			
T1	2.2 ± 0.9	1.7 ± 1.5	0.142
T3	1.3 ± 1.6	3.2 ± 1.4	<0.001
P-value^2^	0.031	0.001	
Pain interference in the last week (7)			
Enjoyment of life			
T1	2.0 ± 0.8	1.7 ± 1.9	0.289
T3	1.1 ± 1.6	3.0 ± 1.5	<0.001
P-value^2^	0.005	0.653	

*∗*Mann-Whitney U test was used to compare 2 groups

*∗* Generalized Estimating Equation (GEE) model was used to compare T1-T3

P-value^1^: Comparison of data between HBOT group and Control group

P-value^2^: Comparison of data between T1 and T3.

## Data Availability

The data used to support the findings of this study are available from the corresponding author upon request.
